# Development of specific instrumentalist exercises to improve scapular stabilization—a proof of concept study (New exercises to strengthen musicians – Part I)

**DOI:** 10.3389/fpsyg.2026.1825799

**Published:** 2026-06-24

**Authors:** Céleste Rousseau, Christoff Zalpour, Ju-Yang Chi, Bronwen J. Ackermann

**Affiliations:** 1 Department of Movement and Rehabilitation Science, Faculty of Business, Management, and Social Science, University of Applied Sciences Osnabrück, Osnabrück, Germany; 2 Unité de Recherche UR 20201 REHADAPT, Université Versailles Saint-Quentin – Paris Saclay, Versailles, France; 3 USR 3608 République des Savoirs, École Normale Supérieure, Paris, France; 4 School of Medical Sciences, Faculty of Medicine and Health, University of Sydney, Camperdown, NSW, Australia

**Keywords:** electromyography, exercises, motor control, musical performance, musicians, scapular stabilization

## Abstract

**Introduction:**

Shoulder pain is a common problem occurring in musicians, with impairments in motor control and poor scapular stabilization considered potential causative risk factors. Despite research highlighting the importance of scapula exercises for managing shoulder pain, there are minimal guidelines for the best exercises to apply in musicians. The aim of this study is to evaluate the effect of purpose-designed musician scapula stabilization exercises on the activation patterns of shoulder muscles.

**Method:**

In this quasi-experimental pilot study, five task-specific exercises were designed based on upper string players’ movements while playing to activate scapula musculature, and were compared with existing exercises, using surface electromyography on key stabilizing and on forearm flexor muscles.

**Results:**

Exercises successfully activated the selected scapula stabilizing muscles, particularly the bilateral exercises. For unilateral exercises, higher activations for middle and lower trapezius occurred during exercises involving pulling actions. However, these unilateral exercises also showed effects on the contralateral muscles, indicating bilateral potential benefits.

**Conclusion:**

The musical-instrument-specific scapula exercises were shown in this pilot study to recruit the targeted muscles, with bilateral exercises having more effect than unilateral exercises. As this was a small pilot study, the value of unilateral exercises, and their contralateral effects, may still be apparent, but larger sample size are needed. Further research should investigate effects on pain or function.

## 1 Introduction

Playing-related musculoskeletal disorders (PRMDs) were fist defined in professional musicians in 1998 (Zaza et al., 1998): since then between 26 and 85% of musicians have been reported to have suffer from PRMDs (Silva et al., 2015). These reported injuries are usually considered to be multifactorial, with commonly reported risk factors including “poor” posture during playing and improper muscle balance (Steinmetz et al., 2010). One of the most commonly reported affected body regions is the shoulder (Kok et al., 2015), and these typically do not recover well (Ackermann et al., 2012). In the literature to date, minimal functional guidelines exist regarding the best rehabilitation approaches for upper limb PRMDs in musicians (Chan et al., 2014a; Chan et al., 2013). This is in stark contrast to the athletic population, whereby scapula stabilization and shoulder muscle balance exercises are reported to be effective in managing shoulder pain and injury (Cools et al., 2016). As for athletes, from a contemporary motor control and dynamic systems perspective, skilled musical performance might be considered as a continuous interaction between the individual, planning a task, and the environment. Efficient upper-limb performance depends not only on isolated muscle strength, but also on the coordination of proximal and distal body segments throughout task execution (Sciascia and Kibler, 2022). Current theories of motor planning and sensorimotor learning further suggest that movement organization relies on task-dependent motor strategies and continuous adaptation processes shaped by sensory feedback and performance demands (Kim et al., 2021). Within this framework, scapular stabilization may contribute to the development of efficient upper-limb motor patterns by providing proximal control required for fine distal motor performance during instrumental playing (Sciascia and Kibler, 2022).

The scapula provides the ‘ground’ for all arm movements, as the glenoid fossa of the scapula provides the only attachment site for the humerus to the body. The scapula itself needs to be both mobile and stable to provide this platform role for the humerus to move on throughout the extensive range of motion of the shoulder (Mottram, 1997). The scapula itself has only one bony joint attaching it to the body—the acromioclavicular joint—with it otherwise supported in its position on the posterior chest wall (the so-called scapula-thoracic joint) by a large number of muscular attachments (Mottram et al., 2009; Nijs et al., 2005). For fine motor control of the distal upper extremity, as is typically required in music performance, the scapula stabilizing musculature is considered to play an extremely important role in the kinetic chain of support required for the athletic type activities of the upper limbs in musicians (Sciascia and Kibler, 2022). Some of the most important muscles are reported to be trapezius (upper, middle and lower) and serratus anterior (Kibler et al., 2008; Mottram et al., 2009) and their insufficiency or weakness could at least partially explain scapular dyskinesis (Sciascia and Kibler, 2022).

As scapula dysfunction may lead to altered upper limb motor control (Sciascia and Kibler, 2022), biofeedback can be a useful tool to enhance functional use of scapula stabilizer muscles during a specific task (ie. playing an instrument), using principles of optimal feedback control theory, to integrate changes into the target movements (Kim et al., 2021). For musicians, shoulder and scapula demands are considered important to support the upper limb actions while playing, as briefly highlighted in Table 1. Since many musicians are reported to show a lack of scapular motor control or scapular dyskinesis (Frizziero et al., 2018; Silva et al., 2018; Tawde et al., 2016), training the control of their scapula stabilizer muscles in as task-specific a manner as possible may be an effective intervention, both to reduce PRMDs and to support the playing action.

**Table 1:** Shoulder and scapula positioning demands in different instruments.

Instrument	Piano	Violin	Guitar	Flute
Left scapula	Light controlled protraction	Controlled protraction and elevation (Tawde et al., 2016)	Light controlled protraction	Controlled protraction and elevation
Right scapula	Light controlled protraction	Controlled protraction and elevation (Tawde et al., 2016)	Protraction and elevation	Controlled protraction
Left shoulder	Light flexion, movements of light abduction and adduction	Flexion, light abduction and important external rotation (Shan and Visentin, 2003)	Flexion and external rotation, movements of abduction	Flexion, light abduction, external rotation.
Right shoulder	Light flexion, movements of light abduction and adduction	Flexion, light to important abduction, light controlled internal rotation (Tawde et al., 2016)	Abduction, light controlled internal rotation	Light extension, abduction, external rotation.
Shoulder pain	44% (Wood, 2014)	30% (Ackermann et al., 2012)	15–20% (Ackermann et al., 2012; Fjellman-Wiklund and Chesky, 2006)	30% (Spence, 2001)

According to muscle strengthening and motor control literature, movements should be trained in a task-specific manner (Hubbard et al., 2009). Muscle biofeedback, using such tools as surface electromyography, can provide an effective method of measuring the muscle activation pattern effects of training interventions (Saeterbakken et al., 2016).

Given the need for task-specific strengthening, traditional methods of increasing scapular motor control in the clinical setting may not be readily transferable into musical instrument playing without modifications. However, as there is a lack of available evidence on the best methods of scapula retraining to apply to musical performance, we can potentially mimic approaches used for athletes’ rehabilitation (Cools et al., 2016). This could be done by creating functional exercises for musicians that may better help them recover from shoulder and upper limb disorders, and even to potentially reduce the risk of incurring such problems. The development of task-specific exercises which could mimic instrumental gesture may better allow musicians to translate improved muscle activation into their performance, with less internal focus that might distract from performance in expert musicians (Duke et al., 2011), and more precisely in violinists (Allingham and Wöllner, 2022).

Using a theoretically informed rehabilitation framework, this paper aims to bridge the gap between motor control and biomechanical principles and clinically applicable interventions for musicians (Hudon et al., 2015). More specifically, this pilot study presents the development of task-specific scapular stabilization exercises designed for guitarists, violinists, flutists, and pianists, which may be readily implemented by physiotherapists and other healthcare professionals working with musicians.

The aims of this study are to:

1. Develop a series of task-specific scapula activation exercises based on the shoulder position and movements involved when playing some common instruments.
2. Pilot test the developed exercises using surface electromyography (sEMG) to ascertain if desired muscles are activated, to provide a proof-of-concept rationale for using these exercises as a training tool for future exercise intervention studies.

## 2 Methods

The inclusion criteria to the study were that participants were aged between 18 and 34 (Kok et al., 2015) and were able to speak English fluently. The study was approved by The University of Sydney Human Research Ethics Committee (HREC: 2018/219). The pilot study took place in the biomechanics laboratory of the University of Sydney. After reading an information sheet, participants signed an informed consent form before commencing the study. A sample of 4 musician participants (pianist, guitarist, violinist and flutist) agreed to participate in this pilot study. All musicians were amateur musicians, playing their instrument for about 10 years, with no regular practice. All participants reported no pain in the neck and upper limbs.

The muscles selected for sEMG included those felt to play a role in scapular stabilization as well as the deltoid to measure activity in relation to arm elevation. These shoulder muscles included upper—UT, middle—MT— and lower trapezius—LT, anterior deltoid—AD, and serratus anterior—SA (Kibler et al., 2008; Nijs et al., 2005). Muscle activity was also recorded from the Flexor Carpi Ulnaris (FCU) as previous research suggests potential proximal and distal muscle activity relationships in musicians (McCrary and Ackermann, 2016). SENIAM recommendations (Hermens et al., 2000) were used to guide the sEMG-protocol for the shoulder muscles and Criswell for the other electrode placements (Criswell, 2010).

Two Ag/AgCl surface electrodes (Red Dot, 2,258; 3 M, Sydney, NSW, Australia) were placed 2 cm apart in parallel with the muscle fibers of each selected muscles. Fixomull hypoallergenic adhesive tape (Smith & Nephew, North Ryde, NSW, Australia) was applied as necessary to prevent movement of electrodes during trials. Electrodes were connected to wireless sEMG sensors (TELEmyo DTS EMG sensors; Noraxon, Scottsdale, AZ, USA— ~ 14 gm, 3.4 × 2.4 × 1.4 cm) and amplified with a 1st-order band-pass filter, bandwidth of 10–500 Hz, gain of 500, input impedance >100 MΩ, and common mode rejection >100 dB. The amplified signals were transmitted to a 16-bit resolution receiver (TELEmyo DTS belt receiver, Noraxon) and saved to a computer at a rate of 1,500 Hz using MR3 software (ver 3.6.20; Noraxon). Electrodes were also put in play position to avoid the skin slidings. The movements have been also followed by a 3D-motion capture (Noraxon®).

In all tests, participants were instructed to contract isometrically with maximum effort for 3 s against resistance provided by the same researcher. Each Maximum Voluntary Contraction (MVC) was repeated 3 times for 5 s, separated by 1-min rest period. The following 5 MVCs were performed, as per Ginn et al. (2011) protocol: shoulder internal rotation at 90° elbow flexion, abduction at 90° abduction, shoulder flexion at 125° flexion, shoulder extension at 30° abduction, and flexion of the wrist (*flexor carpi ulnaris*). Signal processing was performed with the Noraxon Software. sEMG signals were high-pass filtered at 10 Hz (zero-lag, 8th-order Butterworth), rectified, and then the linear envelope was calculated by low-pass filtering at 3 Hz (zero-lag, 8th-order Butterworth). All signals were visually inspected prior to processing by blinded study personnel. Using the maximum amplitude recorded for each muscle across all MVC tests, the excerpt sEMG signals were then normalized and expressed as %MVC.

Once sEMG electrodes were applied and MVCs recorded, the trial commenced with sEMG recordings captured at each stage—pre, during and post exercise. Participants were asked to play their instrument using an excerpt of their choice, then to do a specific exercise guided by one of two physiotherapists. Before the very first session, the three physiotherapists (B. A., J.-Y. C. and C. R.) who developed the protocol, agreed on standardized instructions in order to keep them the same with all participants. Two physiotherapists (J.-Y. C. and C. R.) were present at every testing. Following completion of the exercise, participants were asked to play their chosen piece again. To minimize bias introduced by fatigue or therapist, and randomization process was used whereby participants selected an exercise and the physiotherapist from pieces of paper in a bag so that they were blinded to the experimental condition. The two pieces of paper selected by the participants determined (i) the exercise and (ii) which physiotherapist researcher would teach the exercise. Participants were encouraged during both playing measurement sessions to be aware of their shoulder blades. In the post-exercise playing activity, participants were additionally encouraged to reproduce the sensations of scapula stabilization that they had worked on during the exercises. Each exercise was first demonstrated by the physiotherapist and then performed by the subject 5 times, each time with a 5 s hold, while muscle activity levels were recorded.

### 2.1 Purpose-designed exercises

Between 3 and 5 exercises per instrument were designed:

- 3 piano-specific exercises: one bilateral, two unilateral ones;
- 5 guitar-specific exercises: one bilateral, four unilateral ones;
- 5 violin-specific exercises: one bilateral, four unilateral ones;
- 5 flute-specific exercises: one bilateral, four unilateral ones.

Each exercise is described and illustrated in Table 2.

**Table 2:** Description of each task-specific exercise.

Instrument	Exercise name	Starting position	Instructions	Photo
Piano	PX1: Bilateral shoulder external rotation (inspired from Chan et al., 2014b)	Standing, forearm in pronation, Theraband® between both wrists.	Stretch the elastic by spreading your wrists horizontally, as if you were playing the piano and open your chest, feeling behind your back your shoulder blade muscle working.	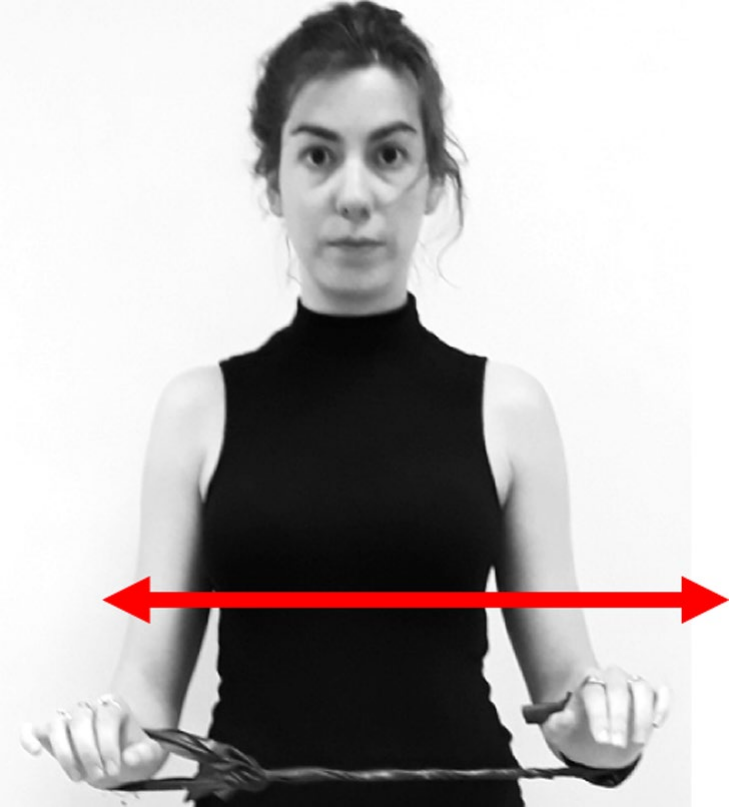
Piano	PX2 (R) & PX3 (L): Abduction of the shoulder	Standing, forearm in pronation, Theraband® around one wrist, fifth finger closer to the door.	Elastic stretched, move your hand in front of you, as if you were playing the piano, following a line in front of you on the wall. Open your chest when your hand goes far from your body, feel your shoulder blade muscles and close your chest Feel also your body weight moving from one leg to the other one.	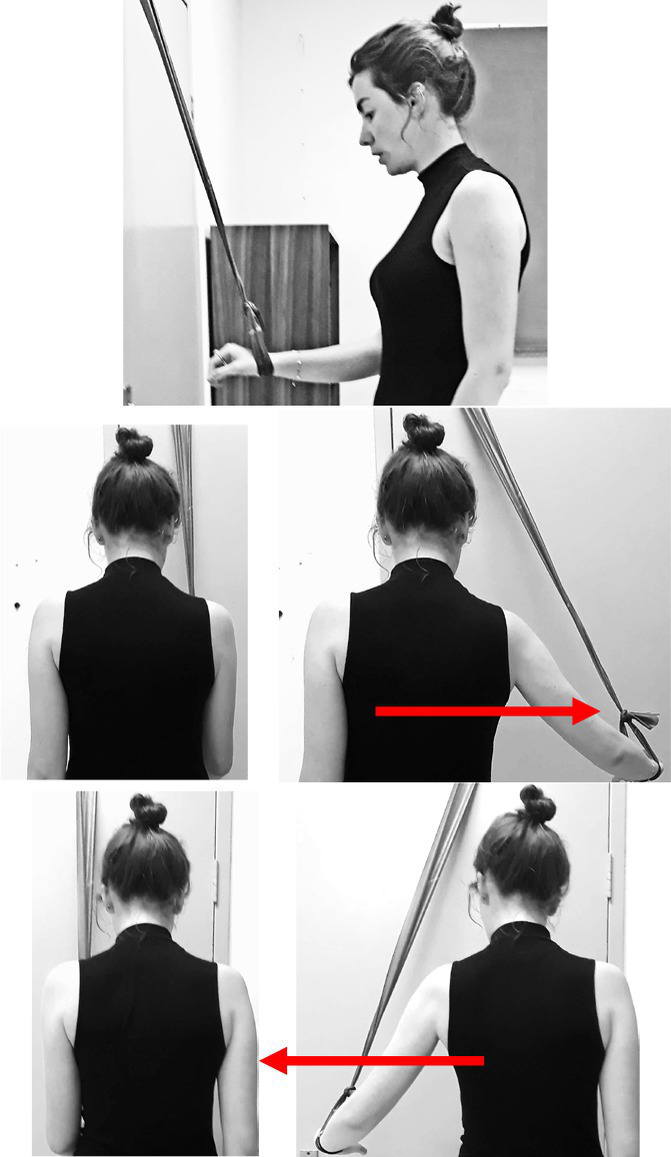
Guitar	GX1: Bilateral shoulder external rotation (inspired from Chan et al., 2014b)	Arms placed in the guitar play position, Theraband® between both wrists.	Stretch the elastic by spreading your wrists with a movement of your right upper limb, in position of playing the guitar, feeling your shoulder blade muscles and opening your chest.	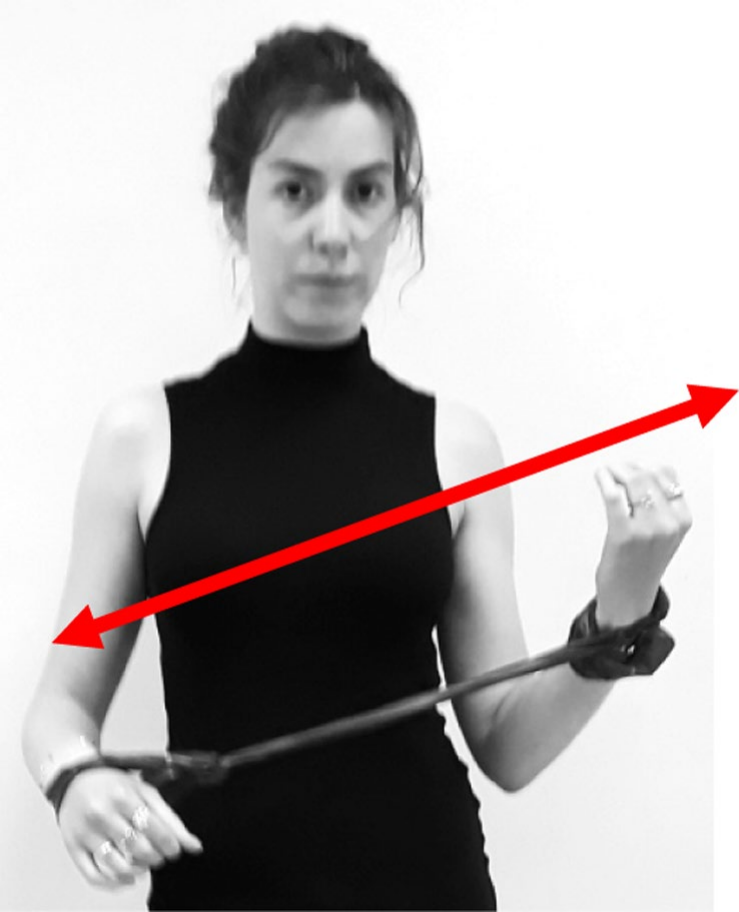
Guitar	GX2: Left upper limb: Theraband® fixed from the behind	Arms placed in the guitar play position, Theraband® around one wrist, fixed from behind.	Stretch the elastic by spreading your wrist from the wall and move your hand as if you were playing the guitar upward and downward, along the guitar neck, with opening your chest and feeling your shoulder blade muscles.	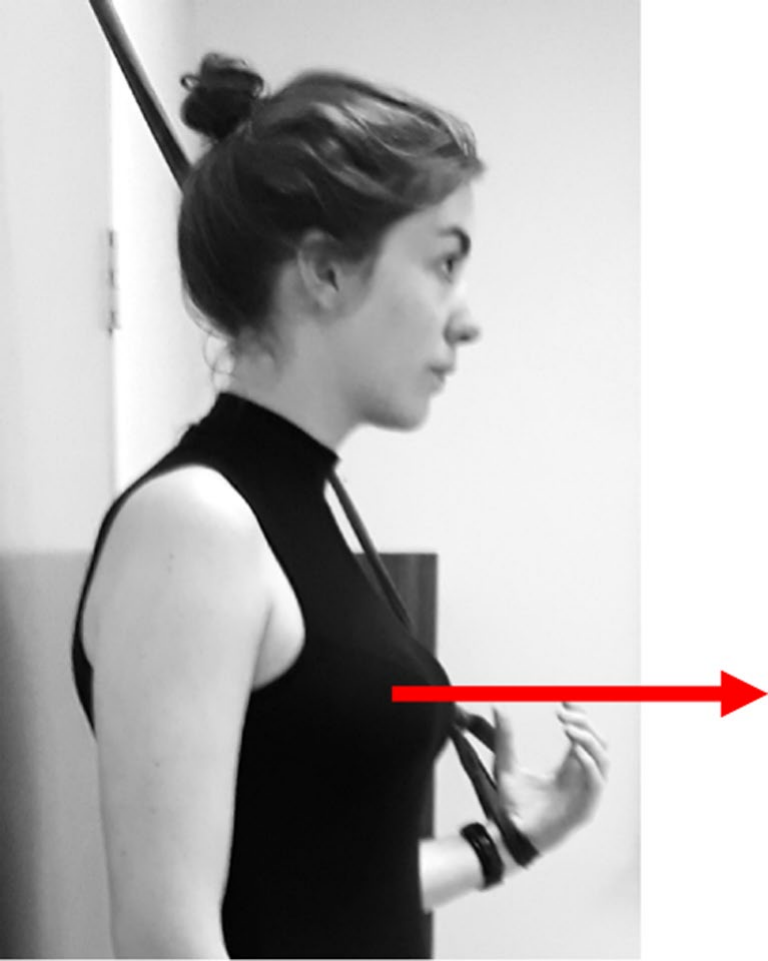
Guitar	GX3: Left upper limb, Theraband® fixed from the front	Arms placed in the guitar play position, Theraband® around one wrist fixed on the front.	Stretch the elastic by spreading your wrist from the wall and move your hand as if you were playing the guitar upward and downward, along the guitar neck, with opening your chest and feeling your shoulder blade muscles.	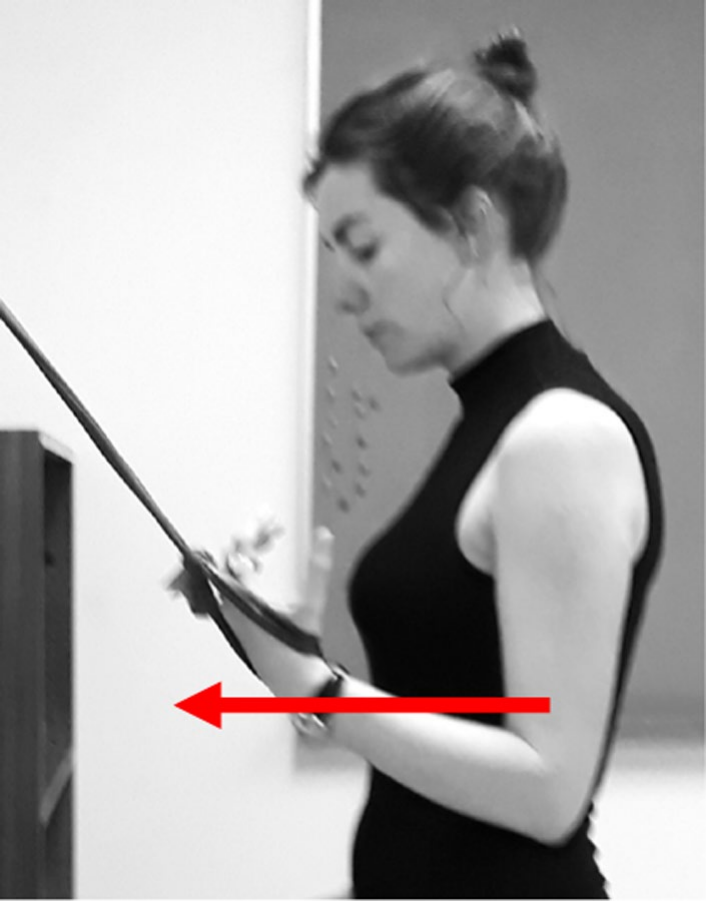
Guitar	GX4: Right upper limb: Theraband® fixed from the behind	Arms placed in the guitar play position, Theraband® around one wrist fixed on the front.	Stretch the elastic by spreading your wrist from the wall and move your hand as if you were playing the guitar, up and down, as if you were touching the strings, with opening your chest and feeling your shoulder blade muscles.	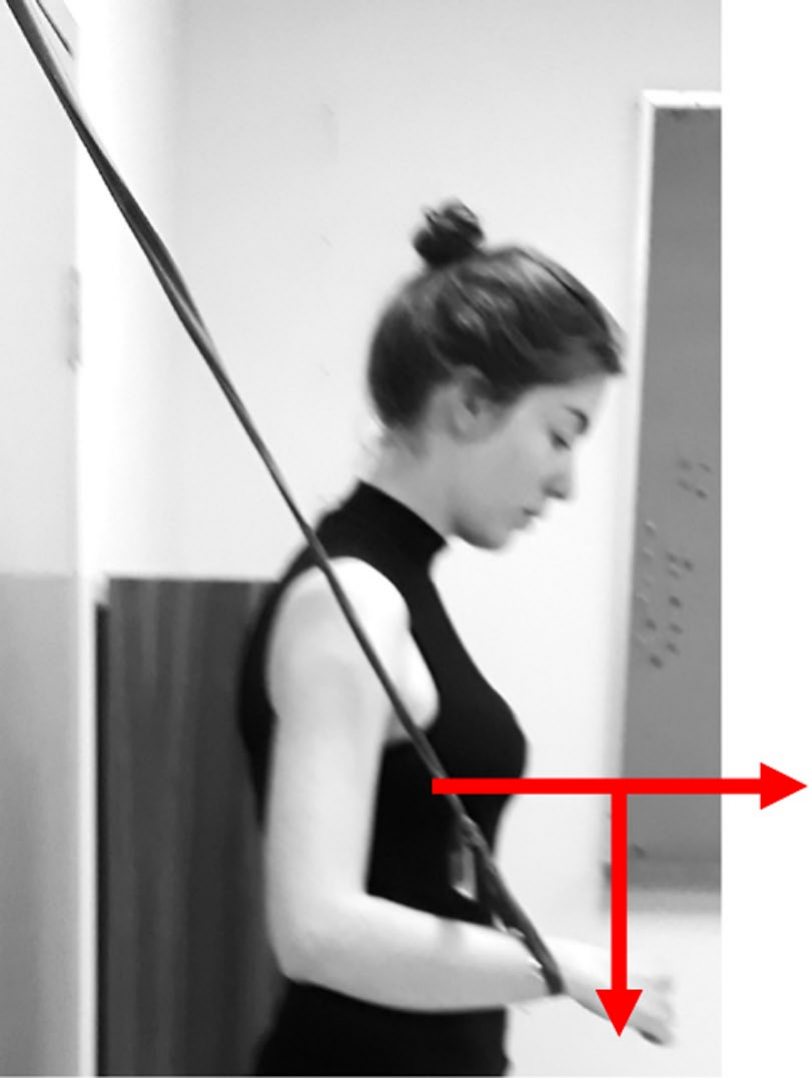
Guitar	GX5: Right upper limb, Theraband® fixed from the front	Arms placed in the guitar play position, Theraband® around one wrist fixed on the front.	Stretch the elastic by spreading your wrist from the wall and move your hand as if you were playing the guitar, up and down, as if you were touching the strings, with opening your chest and feeling your shoulder blade muscles.	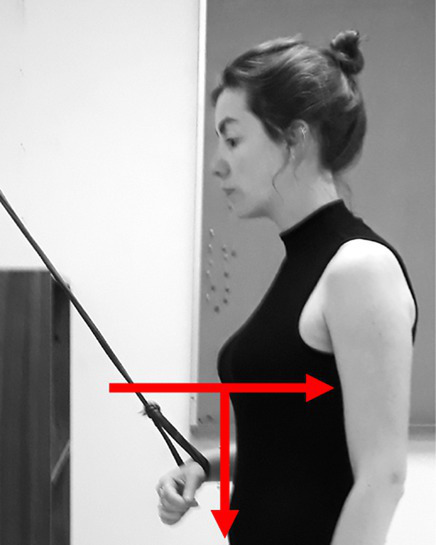
Violin	VX1: Bilateral shoulder external rotation (inspired from Chan et al., 2014b)	Arms placed in the violin play position, Theraband® between both wrists, pen in the bowing hand.	Stretch the elastic by spreading your wrists with a movement of your right upper limb, in position of playing the violin, feeling your shoulder blade muscles and opening your chest.	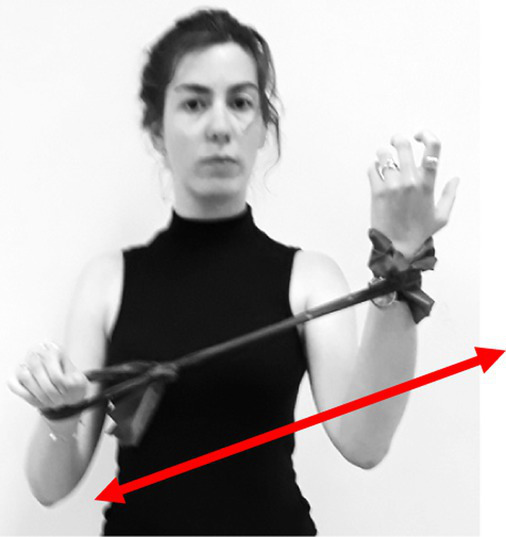
Violin	VX2: Left upper limb: Theraband® fixed from the behind	Arms placed in the violin play position, Theraband® around one wrist, fixed from behind, pen in the bowing hand.	Stretch the elastic by spreading your wrist from the wall forward, as if you were playing along the violin neck, with opening your chest and feeling your shoulder blade muscle.	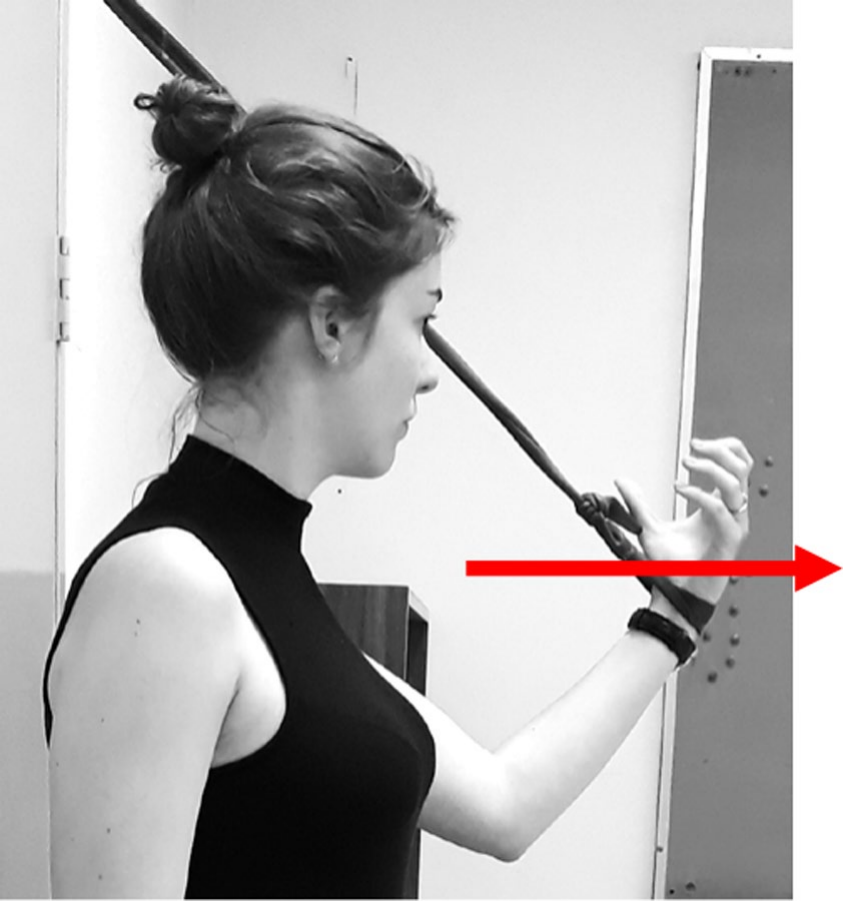
Violin	VX3: Left upper limb, Theraband® fixed from the front	Arms placed in the violin play position, Theraband® around one wrist, fixed on the front, pen in the bowing hand.	Stretch the elastic by spreading your wrist from the wall forward, as if you were playing along the violin neck, with opening your chest and feeling your shoulder blade muscle.	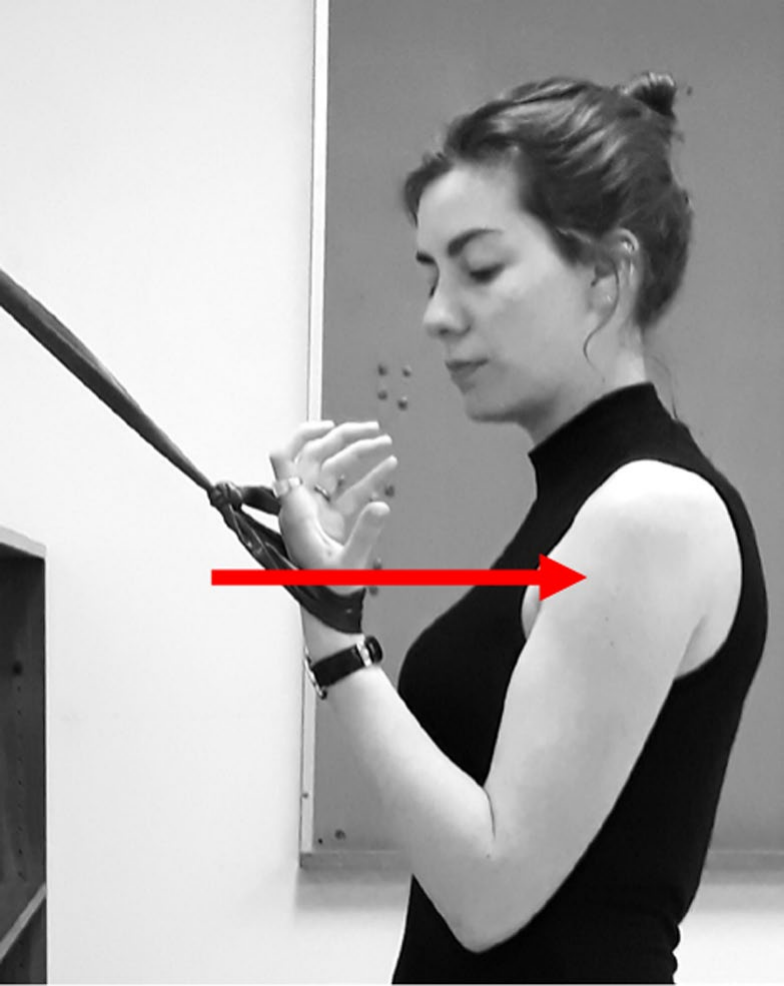
Violin	VX4: Right upper limb: Theraband® fixed from the behind	Arms placed in the violin play position, Theraband® around one wrist, fixed on the front, pen in the bowing hand.	Stretch the elastic by spreading your wrist from the wall, as if you were bowing, with opening your chest and feeling your shoulder blade muscle. You can also feel your weight moving between your both legs.	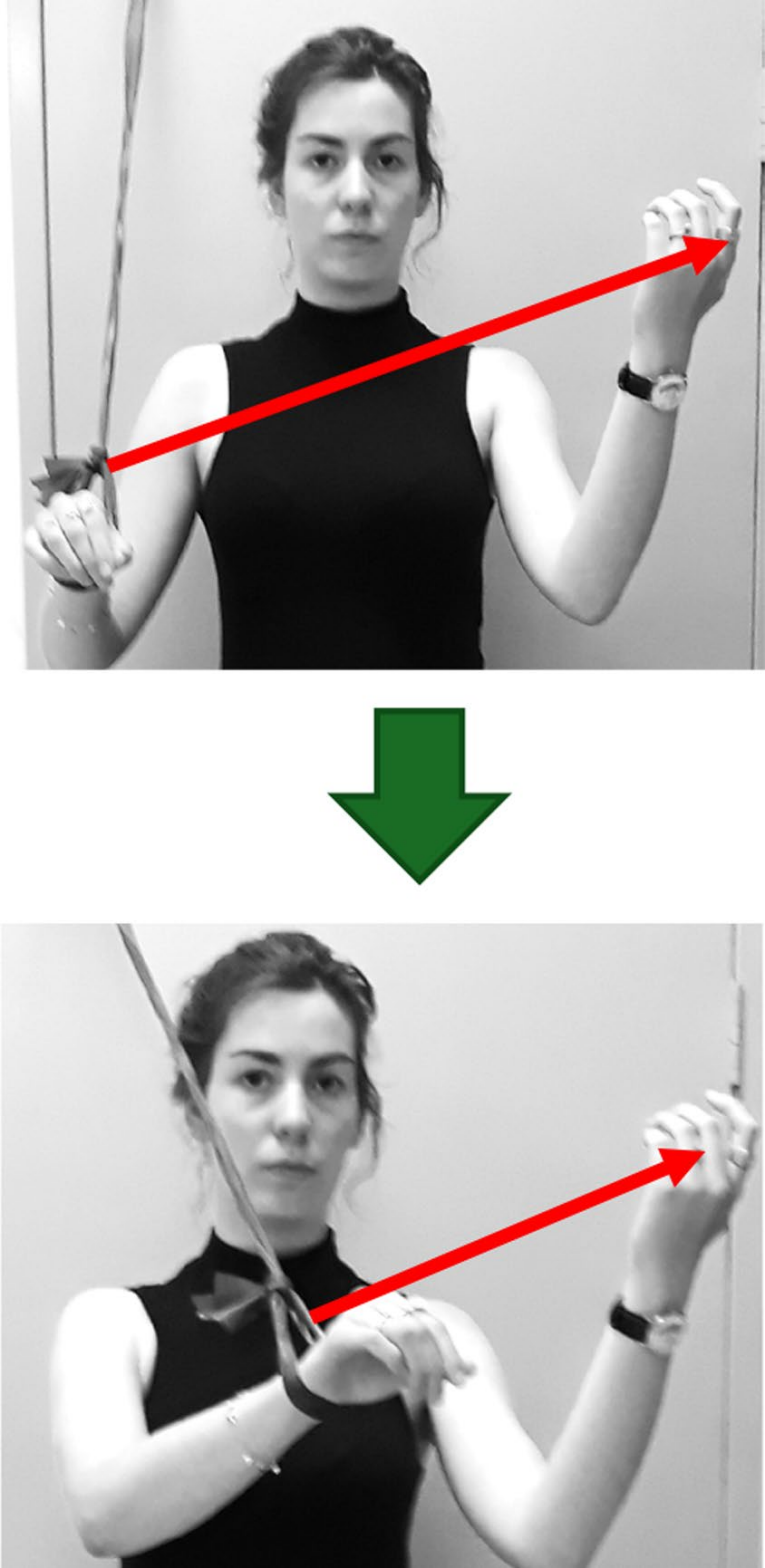
Violin	VX5: Right upper limb, Theraband® fixed from the front	Arms placed in the violin play position, Theraband® around one wrist, fixed on the front, pen in the bowing hand.	Stretch the elastic by spreading your wrist from the wall forward, as if you were bowing, with opening your chest and feeling your shoulder blade muscle. You can also feel your weight moving between your both legs.	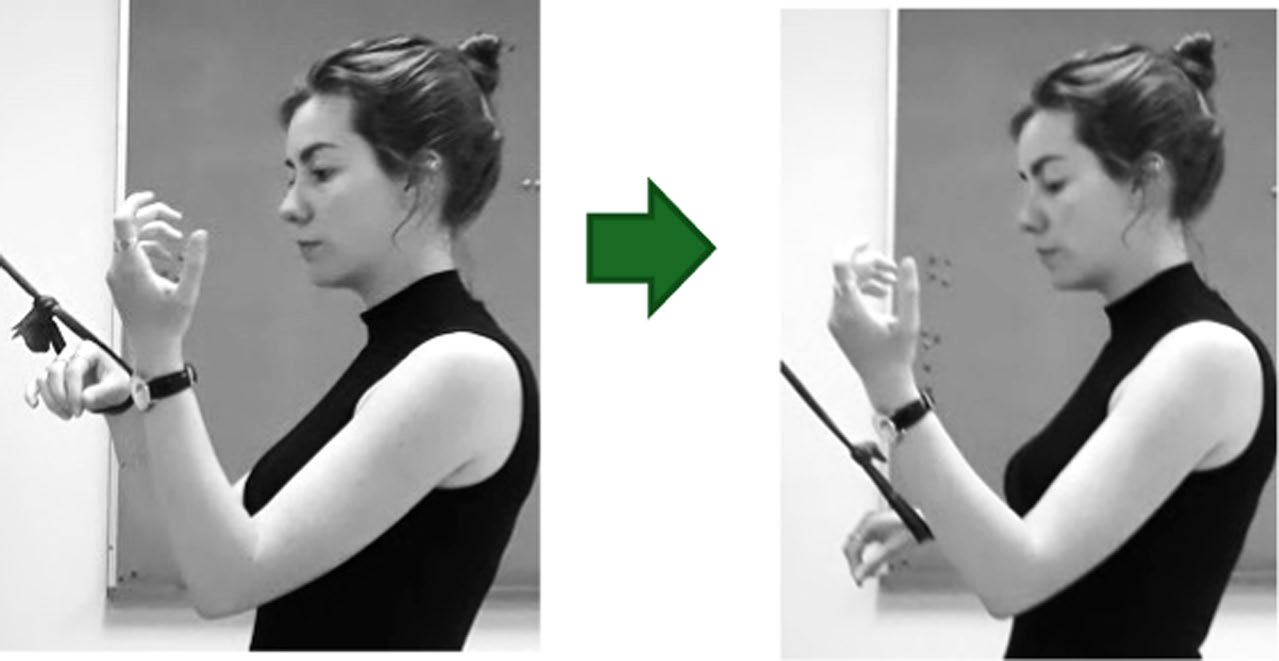
Flute	FX1: Bilateral shoulder external rotation (inspired from Chan et al., 2014b)	Arms placed in the flute play position, Theraband® between both wrists.	Stretch lightly the elastic by spreading your wrists with a movement of your right upper limb, in position of playing the flute, feeling your shoulder blade muscles and opening your chest.	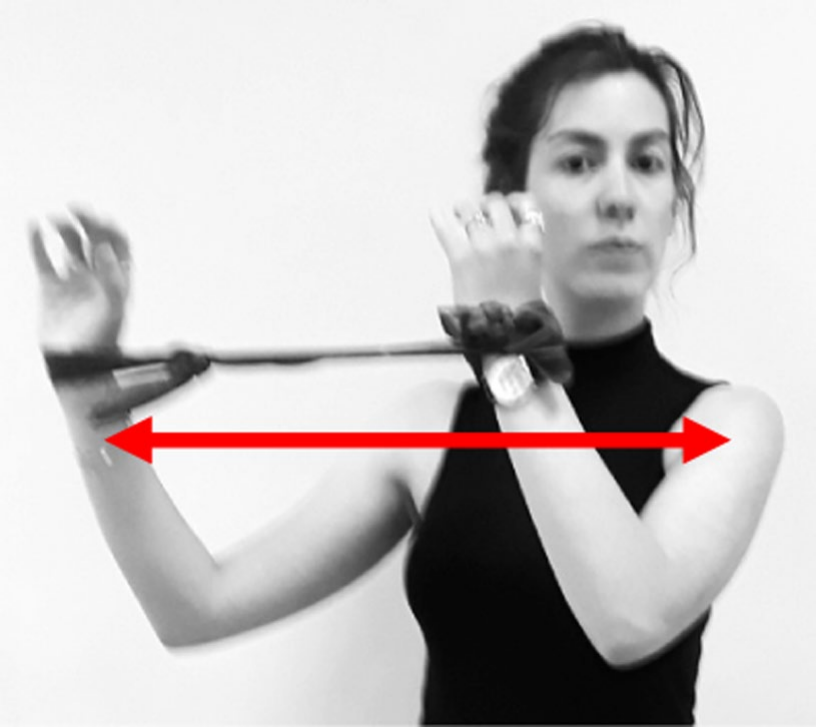
Flute	FX2: Left upper limb: Theraband® fixed from the behind	Arms placed in the flute play position, Theraband® around one wrist, fixed from behind.	Stretch lightly the elastic by spreading your wrist from the wall forward, with opening your chest and feeling your shoulder blade muscles, maintain the position around 5 s, static.	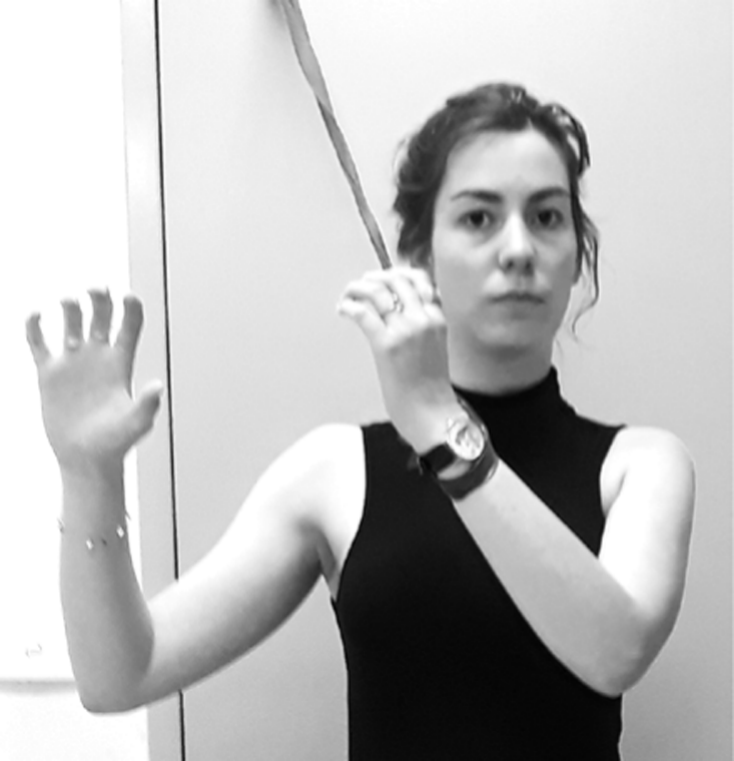
Flute	FX3: Left upper limb, Theraband® fixed from the front	Arms placed in the flute play position, Theraband® around one wrist, fixed on the front.	Stretch lightly the elastic by spreading your wrist from the wall, as if you were bringing the flute to your mouth to play, with opening your chest and feeling your shoulder blade muscles, maintain the position around 5 s, static.	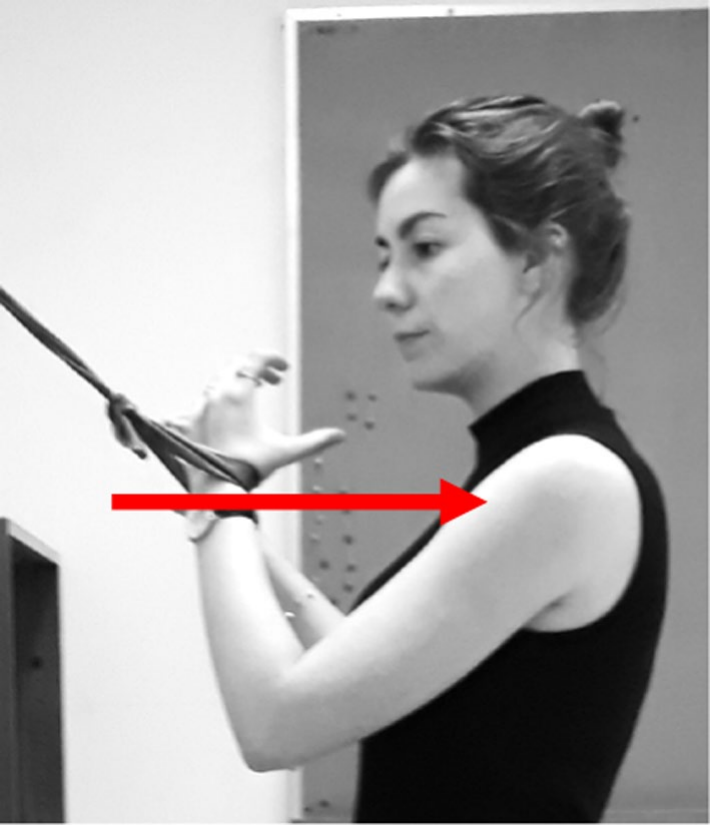
Flute	FX4: Right upper limb: Theraband® fixed from the behind	Arms placed in the flute play position, Theraband® around one wrist, fixed from behind.	Stretch lightly the elastic by spreading your wrist from the wall forward, with opening your chest and feeling your shoulder blade muscles, maintain the position around 5 s, static.	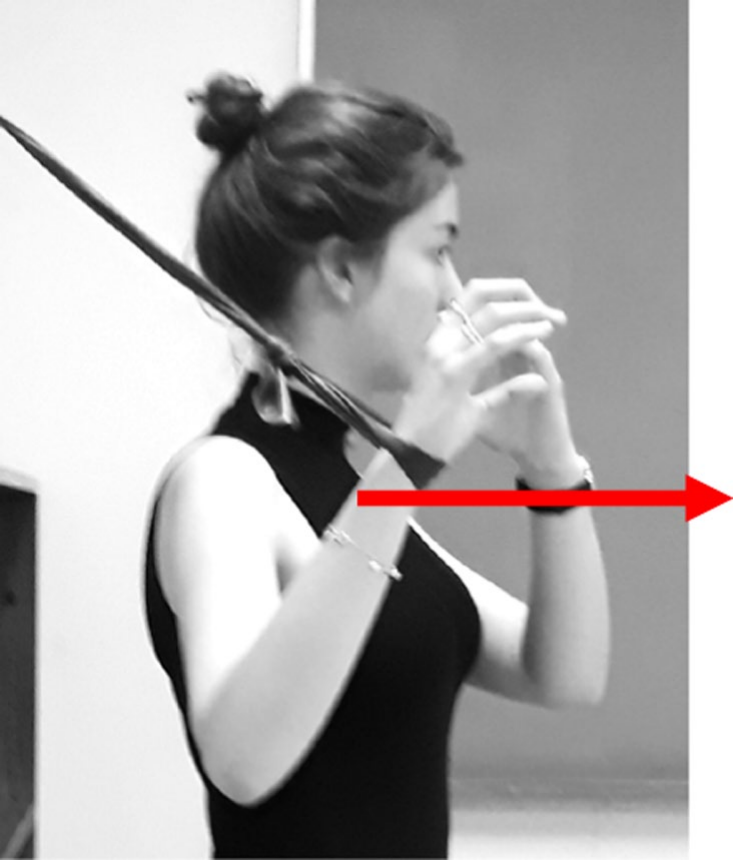
Flute	FX5: Right upper limb, Theraband® fixed from the front	Arms placed in the flute play position, Theraband® around one wrist, fixed on the front.	Stretch lightly the elastic by spreading your wrist from the wall, as if you were bringing the flute to your mouth to play, with opening your chest and feeling your shoulder blade muscles, maintain the position around 5 s, static.	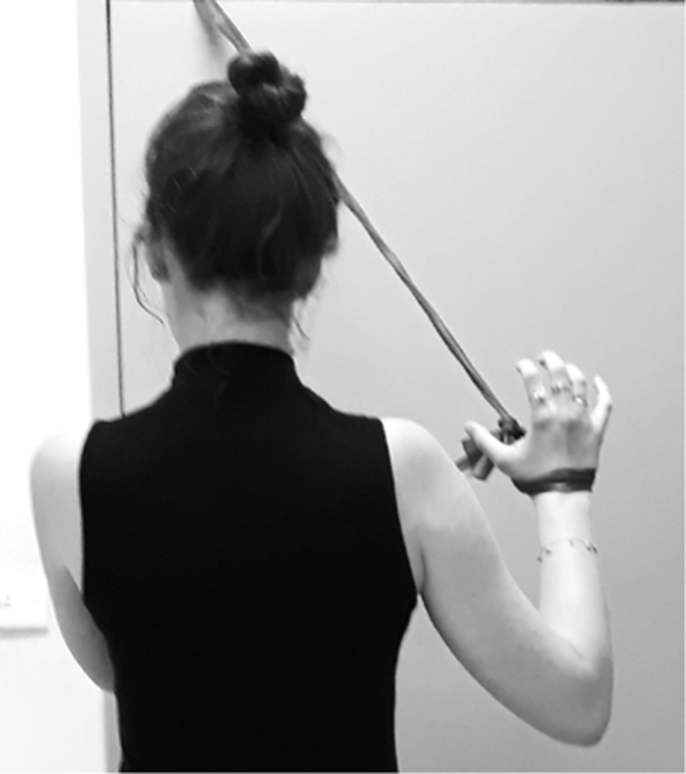

PX, piano exercise; GX, guitar exercise; FX, flute exercise; VX, violin exercise; R, right; L, left.

### 2.2 Statistical analysis

Descriptive statistics were conducted to analyze the percentages of MVCs that muscles reached during the exercises and while playing to evaluate whether one exercise was superior to another in terms of the amount of scapular stabilizers recruitment and activation.

## 3 Results

A sample of 4 participants (2 males: 1 guitarist, 1 violinist; and 2 females: 1 flutist, 1 pianist) completed the pilot trial. The age of the participants was on average 22.8 years (± 4.0), average weight was 73.3 kg (± 9.0) and average height 180 cm (± 4.8). All muscle activation levels during the different exercises are shown in Table 3.

**Table 3:** Muscles activation during exercises.

Exercises		Upper trapezius		Middle trapezius		Lower trapezius		Serratus anterior	
		L (%)	R (%)	L (%)	R (%)	L (%)	R (%)	L (%)	R (%)
Piano	PX1 (B)	9.6 (6.7–10.9)	12.8 (9.9–16.4)	74.3 (54.8–98.5)	67.8 (56.4–84.8)	46.2 (33.9–60.6)	70 (48.9–84.3)	9.8 (7.9–12.8)	15.6 (14.6–16.4)
	PX2 (R)	2.4 (1.1–6.7)	4.2 (2.9–6.3)	3.5 (3.1–4.6)	32.1 (26.2–41.6)	6.7 (5.9–7.3)	24.1 (11.4–33.8)	9.6 (7.9–11.4)	13.3 (9.1–25.4)
	PX3 (L)	19.3 (3.5–38.5)	9.4 (2.3–20.4)	54.4 (37–69.2)	18.4 (14.3–27.7)	22.1 (15.7–31.4)	39.4 (23.8–70.8)	11.6 (6.7–15)	8.5 (7.5–10.2)
Guitar	GX1 (B)	12.8 (8.9–16.1)	10.7 (8.1–12.6)	54.4 (37–69.2)	23.4 (15.4–31)	66.4 (40.9–90.2)	57.5 (30–101.7)	7.7 (3.9–11.5)	3.7 (2–5.3)
	GX2 (L)	8.1 (3.4–11)	3.4 (2.8–4.6)	24.8 (16.4–35.7)	2.0 (1.1–2.7)	18.1 (10.8–33.4)	2.3 (1.8–3.2)	7.6 (6.3–9.7)	0.6 (0.5–0.9)
	GX3 (L)	4.7 (3.9–6.4)	3.8 (3.3–5.2)	3.4 (2.6–4.3)	2.5 (1.6–5.1)	18.1 (7.9–25.6)	1.9 (1.7–2.3)	2,6 (2.2–3.2)	1.0 (0.7–1.3)
	GX4 (R)	2.4 (2–2.7)	2.4 (1.9–3.4)	7.2 (5.3–9)	5.7 (4.2–8)	17.1 (12.6–21.7)	12.2 (8.8–19.1)	1,6 (1.5–1.6)	1.5 (0.9–3.3)
	GX5 (R)	1.6 (1.3–2)	5.4 (3.4–8.9)	3.3 (2.3–4.8)	13.1 (6.6–18.6)	18.7 (8.3–33.8)	24.0 (14.6–33.8)	2.3 (1.8–3.1)	2.6 (1.9–3.3)
Violin	VX1 (B)	6.5 (5.3–8.5)	5.0 (3.6–6.2)	33.7 (30.7–38.6)	25.0 (23.1–26.5)	83.7 (73.7–94.6)	64.6 (53–85.4)	14.4 (10.9–20.2)	5.9 (4.9–7.4)
	VX2 (L)	0.8 (0.6–1.3)	0.9 (0.6–1.8)	8.8 (5.3–12.9)	3.2 (2.8–3.8)	51.9 (32–75)	16.3 (10.6–27.2)	17.7 (10.8–22.7)	3.0 (2.4–4.2)
	VX3 (L)	0.9 (0.6–1.3)	0.6 (0.5–0.6)	23.1 (18–27.3)	3.4 (2.8–4.3)	63.6 (50.8–73.5)	17.9 (13.7–21.3)	7.5 (3.5–11.7)	3.4 (2.5–3.9)
	VX4 (R)	3.1 (2.4–3.5)	3.2 (2.7–4.5)	4.9 (4.1–5.8)	7.6 (5.7–9.6)	30.7 (26.8–38.3)	80.1 (72.8–83.7)	10.7 (9.5–12.1)	8.9 (7.3–14.3)
	VX5 (R)	1,4 (0.8–2.7)	3,1 (2–6.1)	10,9 (8.9–12.8)	23,6 (12.7–36.4)	50,5 (47.5–53.7)	91,4 (82.4–103.1)	13,3 (11.9–14.2)	9,2 (7.2–11.9)
Flute	FX1 (B)	4.0 (1.9–6.4)	5.6 (3.5–11.3)	22.8 (11.4–31.5)	28.1 (11.3–41.7)	51.3 (36.1–62.8)	28.6 (25.5–30.4)	6.8 (4–8.6)	16.4 (14.6–20)
	FX2 (L)	2.3 (2.1–2.6)	3.2 (2.6–3.5)	23.6 (12.2–33.5)	23.6 (12.7–36.4)	50.7 (38.3–61.9)	33.6 (24.5–41.1)	10.8 (7.2–15.8)	35.0 (24.4–42.9)
	FX3 (L)	3.7 (1.6–6)	5.2 (4.1–6.3)	40.1 (25.5–51.5)	24.4 (13.7–38.2)	62.6 (56.1–67.8)	30.0 (22–35.4)	6.9 (4.9–14)	15.5 (11.1–22.3)
	FX4 (R)	1.9 (1.5–3.1)	1.8 (1.6–2.1)	15.4 (12.5–19.7)	20.9 (14.6–26.6)	41.9 (35.5–50.3)	39.9 (29.7–47.2)	7.7 (6.9–8.8)	14.7 (14–16)
	FX5 (R)	1.8 (1.6–2.1)	1.9 (1.5–3.1)	31.5 (18.9–50.2)	27.7 (16.1–36.9)	56.3 (44.2–61.7)	29.2 (21.2–34.6)	6.5 (5–9)	19.3 (14.5–28.4)

Blue: Activation between 0 and 25%MVC, Green: Activation between 25 and 50%MVC, Yellow: Activation between 50 and 75%MVC, Orange: Activation between 75 and 100%MVC. PX, piano exercise; GX, guitar exercise; FX, flute exercise; VX, violin exercise; R, right; L, left; B, bilateral.

In the series of exercises designed for the piano, all the purpose-designed exercises resulted in activation of the upper trapezius, ranging between 2.4 and 19.3% of the MVC. The highest activation levels of both middle and lower trapezius muscles occurred with the bilateral exercise (PX1: MT: 67.8% right, 74.3% left, LT: 70% right, 46.2% left). For the serratus anterior, activation levels ranged between 8.5 and 15.6%.

In the exercises designed for the guitar, the upper trapezius was activated between 1.5 and 12.8%. The highest activation levels of both middle and lower trapezius were again achieved when performing the bilateral exercise (GX1: MT: 54.4% left, 23.4% right, LT: 57.5% right, 66.4% left). Additionally, the left MT and LT were more activated during the 2nd exercise (GX2: MT 24.8% left; LT: 18.1% left) than the 3rd (GX3: MT 3.4% left; LT: 18.1% left) and the right MT and LT muscles were more activated during the 5th exercise (GX5: MT 13.1% right; LT: 24% right) than during the 4th (MT 5.7% right; LT: 12.2% right). The range of serratus anterior muscle activation was low for all exercises—between 0.6 and 7.7% of MVC.

For the violin, the upper trapezius muscle was lightly activated between 0.6 and 6.5% of its MVC across all exercises. The highest activation levels of the right and left middle and left lower trapezius occurred with the bilateral exercise (VX1: MT: 25% right, 33.7% left, LT: 64.6% right, 83.7% left). Left middle and lower trapezius were more activated during the 3rd (X3: MT 23.1% left; LT: 63.6% left) than during the 2^nd^ exercise (VX2: MT 8.8% left; LT: 51.9% left) while the right ones are more activated during the 5^th^ exercise (VX5: MT 23.6% right; LT: 91.4% right) than during the 4^th^ (VX4: MT 7.6% right; LT: 80.1% right). The serratus anterior muscle was activated between 3 and 17.7% of the MVC, with higher levels occurring in the exercises VX1 and VX2.

Finally, in the exercise program designed for the flute, the upper trapezius muscle was only lightly activated, ranging between 1.8 and 5.6% of the MVC. The highest activation levels for lower trapezius on the right side occurred during the fourth exercise (Flute X4: 39.9%) and on the left side during the 3^rd^ exercise (Flute X3: 62.6%). For the middle trapezius, highest activation levels occurred on the right side during the first exercise (Flute X1: 28.1%) and the fifth (Flute X5: 27.7%) and on the left side during the third exercise (elastic fixed from the front: 40.1%). Serratus anterior is lightly activated (between 6.5 and 19.3%), but activation reaches a peak on the right side during the second (elastic fixed from behind: 35%).

All muscles activation levels while playing are reported in Table 4.

**Table 4:** Muscles activation while playing.

Muscles		Upper trapezius		Middle trapezius		Lower trapezius		Anterior deltoid		FCU		Serratus anterior	
		L (%)	R (%)	L (%)	R (%)	L (%)	R (%)	L (%)	R (%)	L (%)	R (%)	L (%)	R (%)
Piano	T1	1.8	3.5	6.0	10.3	3.6	9.3	3.3	1.5	9.4	7.7	2.3	2.8
	T2 (after PX2)	2.4	4.8	4.3	7.4	3.6	8.0	5.6	1.5	11.2	9.8	2.3	2.7
	T3 (after PX1)	3.3	5.9	5.1	8.2	4.9	11.6	6.6	2.8	14.6	12.3	2.8	3.7
	T4 (after PX3)	3.1	5.4	5.0	9.6	5.0	10.6	6.3	2.5	12.9	11.7	2.8	3.5
Guitar	T1	2.3	7.6	7.4	3.1	1.2	0.8	0.5	2.2	3.9	10.7	1.0	0.7
	T2 (after GX1)	3.2	5.4	7.2	3.2	1.7	1.5	0.6	0.6	6.4	12.2	0.9	0.6
	T3 (after GX4)	3.3	5.3	6.6	4.5	1.1	1.5	0.5	0.7	4.4	6.9	0.8	0.5
	T4 (after GX2)	3.4	5.1	6.4	2.9	2.2	1.4	0.6	0.8	3.6	7.7	0.9	0.6
	T5 (after GX5)	2.4	3.3	5.9	5.1	2.0	9.6	0.5	0.6	4.6	6.1	1.0	0.7
	T6 (after GX3)	2.2	5.0	6.9	3.2	3.2	1.4	0.6	0.8	4.7	8.0	1.0	0.6
Violin	T1	3.0	4.2	2.7	1.8	6.6	6.2	2.7	3.0	4.6	0.9	4.5	2.9
	T2 (after VX1)	5.3	3.9	3.1	1.6	4.9	4.7	3.1	2.7	4.6	0.9	4.5	3.3
	T3 (after VX4)	5.4	4.3	3.4	1.5	8.2	3.7	2.8	2.9	4.7	0.8	4.7	3.5
	T4 (after VX5)	5.3	4.5	3.2	1.5	8.7	4.4	2.9	3.0	4.5	0.8	5.0	3.6
	T5 (after VX3)	5.8	4.1	3.2	1.5	3.7	3.9	2.9	2.5	4.5	0.9	5.2	3.6
	T6 (after VX2)	5.7	4.1	3.3	1.6	9.1	5.8	2.7	2.8	4.7	0.8	4.6	3.7
Flute	T1	3.8	5.9	2.8	3.4	9.4	3.3	12.1	9.7	2.8	3.6	8.1	17.6
	T2 (after FX3)	2.1	5.3	2.5	3.3	7.4	3.2	12.9	9.6	2.4	4.9	8.2	19.0
	T3 (after FX2)	1.8	5.4	2.6	3.3	4.5	3.6	12.0	9.6	1.8	6.0	8.5	20.7
	T4 (after FX5)	2.5	5.4	2.6	3.2	5.2	2.4	13.2	10.6	2.2	6.0	8.9	23.2
	T5 (after FX4)	2.5	5.5	2.4	3.1	4.0	2.7	13.0	11.4	3.3	4.7	8.7	20.2
	T6 (after FX1)	4.9	6.0	2.7	3.2	2.9	2.1	13.2	10.7	2.1	7.6	9.4	24.1

PX, piano exercise; GX, guitar exercise; FX, flute exercise; VX, violin exercise; R, right; L, left; B, bilateral; FCU, flexor carpi ulnaris.

While playing the piano, a slight global increase after completing the exercises has been observed between T1 and T4 of both FCU and both upper trapezius muscles. Overall, the highest mean values were obtained after the bilateral exercises (PX1) for upper and lower trapezius, serratus anterior and FCU.

Contrary to the piano playing results, no pattern was observed in the sEMG data during the guitar play: greatest mean values are mainly observed at T1 for several muscles. For both FCU, right UT, left SA and both AD, sEMG activations remained almost the same between T1 and T6.

As during the guitar play, no pattern has been strongly noticed from the sEMG data while playing the violin. Global light increase is observed for left UT and right SA. For both FCU, left UT, right SA and both AD, sEMG activation remained almost the same between T1 and T6. Concerning the lower trapezius, a noticeable increase has been observed at T6 after the left exercise of pushing against the elastic fixed from the behind (VX2).

While playing the flute, MT and LT are the most activated at T1. A major decrease is noticed for the LT between T1 and T6, particularly for the L, when MT, right UT and left FCU remained almost identical. A global increase is also noticed for the left SA and the right FCU. High values for right serratus anterior could be explained by a lack of contraction during the MVC.

There was no difference observed in muscle activation levels in any of the exercises as a consequence of having either of the two different physiotherapists describe and teach the exercises.

## 4 Discussion

### 4.1 Exercises outcomes

This proof-of-concept paper provided useful information detailing how purpose-designed exercises for specific instruments produced relative activation of scapula stabilizer muscles. This pilot data provided a useful first step in developing and testing specific exercises that were to be used later for larger scale exercise intervention trials.

Across all the instrument-task specific exercises developed in this paper, the one that appeared to increase activation levels in the middle and lower trapezius muscles the most occurred when externally rotating both shoulders against the resistance supplied by a thick elastic band (Theraband®). This exercise was inspired from the exercise described by Chan et al. (2014a) for musician shoulder blade strengthening, and similar versions have been widely reported in the literature as useful in shoulder rehabilitation. This bilateral exercise recruited middle and lower trapezius above all other tested muscles in our pilot study. It was slightly modified for each instrument to best mimic the playing position (see Table 2). This was done to enhance the perceived relevance of doing the exercise for their instrumental benefit. This simple adaptation can be included in shoulder rehabilitation programs for musicians where lower and middle trapezius strengthening is desired. In addition, including such an exercise in regular training regimes may act to maintain muscle balance around the shoulder, given the preponderance of shoulder use in front of the body when playing musical instruments, potentially playing a protective role against developing PRMDs. The pattern of increased activation of these muscles may at least in part explain the positive results observed on PRMDs by Chan et al. (2014a). Slight modifications may enhance perceived relevance without negative impact on activation patterns. This may provide important additional motivation to help patient compliance in treatment or prevention programs.

For all unilateral exercises, pulling the elastic when fixed from the front seemed to enhance the activation levels of the lower and middle trapezius muscles compared to pushing the band forwards when fixed from behind. This relates well also to the musical context in that it is logical to perform an exercise that mimics bringing the instrument to oneself than to push it away. As a consequence, the scapular stabilizers are more activated with the third (X3) and the fifth (X5) exercises and less so with the second (X2) and the fourth (X4) ones. Furthermore, it was observed that during the third and fifth exercises where unilateral pulling actions were required, there was also an increase in activation for middle and lower trapezius on the contralateral side.

The forearm musculature (*flexor carpi ulnaris*) activation was measured during playing of all instruments, before and after the exercises. The goal of monitoring forearm muscles was to observe any changes in activation as a consequence of the focus on scapular stabilization. No changes in a short-term were observed.

Concerning the translation of any observable changes in muscle activation levels during playing after the exercises, this was only observable during piano playing. After the bilateral exercise (PX1), an increase in scapular stabilizer activation was observed, particularly in relation to average activation levels of the lower trapezius and serratus anterior. No such pattern was noticeable for flute, guitar and violin playing after the exercises. The lack of change could be attributed to a number of reasons. Firstly, only 5 repetitions of each exercise were performed by a single subject. Secondly, these were amateur rather than professional musicians and perhaps this may have reduced the ability to alter use of scapula muscles. And finally, no strength changes would be expected following a brief series of muscle activation exercises.

### 4.2 Strengths and limitations of the study

This study has several strengths. First, volunteer participants did not have any pain in the upper limbs, which could interfere with exercises or playing their instruments. Secondly, all exercises have been performed with a randomized order to avoid the potential biases caused by fatigue or learning effects. Any overall learning effect should then only reasonably be explained by the improved ability of the participant to feel the shoulder muscle activation as a consequence of the exercise performed. Finally, using two physiotherapist investigators to randomly teach the exercises, suggests that with training, every physiotherapist could teach their musician patient these scapular stabilization exercises.

The major limitation of the study is that this pilot study was specifically conducted with the goal of using a small number of participants on a few instruments to test whether purpose-designed exercises were appropriate and potentially effective to be used in planned larger exercise trials. As such, this process evaluation was felt to be important to optimize potential outcomes of future work using this ‘proof-of-concept’ approach. For this reason, only descriptive data could be presented, as statistical analysis was not possible to definitively conclude the superiority of one exercise compared to other. Instead, trends of activation levels in desired targeted muscles could be observed.

### 4.3 Perspectives

From this pilot testing, future studies aim to explore in more detail whether these changes in scapula muscle activation are measurable in a larger group of skilled musicians, and explore different time frames for implementing such retraining interventions using these exercises. It may also be useful to gather further information, such as perceived effect of these exercises in playing immediately afterwards, to evaluate whether motivation is enhanced by using such task-specific approaches. Furthermore, it could be useful to consider using such an approach to develop modifications of exercises to suit all different orchestra instruments. Finally, it would also be relevant to compare the activation of our task-orientated exercises to the typical shoulder external rotation exercises, often trained in neutral positions such as with the arms by the side with a 90° bent elbow.

### 4.4 Practical implications

The present study has several clinical implications for healthcare professionals working with musicians. First, this pilot study provides preliminary support for the use of task-specific scapular motor control exercises designed to selectively activate relevant stabilizing muscles during instrument-related movements. These findings may also help clinicians develop similarly task-specific rehabilitation approaches for other instrumentalists and for different body regions commonly affected by PRMDs.

Furthermore, adapting rehabilitation principles commonly used in sports medicine to musicians may provide a more functionally relevant and performance-oriented approach for the management of PRMDs.

## 5 Conclusion

In this pilot study, instrument-specific exercises were developed and then tested to evaluate whether desired muscle activity was triggered by comparing of electromyographic data from a range of scapula stabilizing muscles. This preliminary data suggested that bilateral exercises of scapular reached higher activation levels of scapula stabilizers than unilateral ones. Moreover, concerning the unilateral exercises, ones involving ‘pulling’ gestures rather than with ‘pushing’ ones recruited the scapula stabilizers better and had some contralateral scapula muscle activation effect. This data will inform future research trials implementing these exercises in larger populations of musicians.

## Data Availability

The data analyzed in this study is subject to the following licenses/restrictions: data could be made available on request. Requests to access these datasets should be directed to celesterousseau.kine@gmail.com.
